# The predictive value of the FT3/FT4 ratio for the severity of coronary artery disease in patients with acute coronary syndrome

**DOI:** 10.3389/fphys.2025.1696692

**Published:** 2025-12-10

**Authors:** Hui He, Zhen Zhang, Long Xia, Caiyan Cui, Maoling Jiang, Qiao Feng, Xiufen Peng, Jie Feng, Dongyue Jia, Yan Luo, Hanxiong Liu, Li Chang, Lin Cai, Shiqiang Xiong

**Affiliations:** 1 Department of Intensive Care Unit, Jinniu District People’s Hospital, Jinniu Hospital of Sichuan Provincial People’s Hospital, Chengdu, Sichuan, China; 2 Department of Cardiology, The Third People’s Hospital of Chengdu, Affiliated Hospital of Southwest Jiaotong University, Chengdu Cardiovascular Disease Research Institute, Chengdu, Sichuan, China; 3 Department of Emergency Medicine, Sichuan Provincial People’s Hospital, University of Electronic Science and Technology of China, Chengdu, Sichuan, China

**Keywords:** acute coronary syndrome, coronary angiography, syntax score, thyroid hormones, the FT3/FT4 ratio

## Abstract

**Background and Aims:**

Thyroid hormones critically regulate cardiovascular homeostasis, with thyroid dysfunction established as an independent risk factor for coronary artery disease. While free triiodothyronine (FT3) to free thyroxine (FT4) ratio reflects peripheral thyroid hormone conversion efficiency, its prognostic utility for anatomical severity of coronary lesions in acute coronary syndrome (ACS) remains undetermined.

**Methods and Results:**

This observational study enrolled a total of 431 ACS patients who underwent coronary angiography. The anatomical severity of coronary lesions was quantified by SYNTAX score. Patients were stratified into three groups based on tertiles of the FT3/FT4 ratio: T1 (FT3/FT4 ≤0.27, n = 144), T2 (0.27 < FT3/FT4 ≤0.33, n = 144), and T3 (FT3/FT4 >0.33, n = 143). They were further categorized into low-risk (baseline SYNTAX score [bSS] <23) or mid-/high-risk (bSS ≥23) subgroups based on bSS. Compared with the T1 group, patients in the T2 and T3 groups had significantly lower bSS values. Multivariate logistic regression analysis demonstrated that the T3 group had a 53% lower risk of mid-/high-risk SYNTAX scores than the T1 group (odds ratio [OR] 0.470; 95% confidence interval [CI]: 0.227–0.971; P = 0.041). The area under the receiver operating characteristic curve (AUC) for the FT3/FT4 ratio in predicting mid-/high-risk SYNTAX scores was 0.656 (95% CI: 0.587–0.724; P < 0.001). Clinical decision curve analysis confirmed the clinical utility of the FT3/FT4 ratio for this prediction. Additionally, restricted cubic spline analysis revealed a negative dose-response relationship between the FT3/FT4 ratio and SYNTAX scores (non-linear P = 0.017).

**Conclusion:**

Impaired peripheral thyroid hormone (increased of the FT3/FT4 ratio) conversion efficiency was independently associated with increased coronary anatomical complexity in ACS patients.

## Introduction

Thyroid hormones (THs) are critical for maintaining normal cardiovascular physiology (Razvi et al., 2018; Lymvaios et al., 2011; Jabbar et al., 2016; Pingitore et al., 2016). Mounting evidence links abnormal thyroid function to an elevated risk of coronary artery disease (CAD) (Stojković and Žarković, 2020; Manolis et al., 2020), with recent studies extending this association to the severity of coronary lesions. For example, research using the Gensini scoring system to quantify CAD severity has identified an inverse relationship between free triiodothyronine (FT3) levels and the extent of coronary atherosclerosis (Yu et al., 2022; Daswani et al., 2015), confirming that thyroid dysfunction correlates closely with coronary artery damage.

Beyond overt thyroid dysfunction, most individuals first exhibit changes in thyroid sensitivity indices before abnormal thyroid function becomes apparent. Thus, in people with normal thyroid function, early disruptions in thyroid hormone homeostasis can be detected using these indices—with the free triiodothyronine to free thyroxine (FT4) ratio emerging as a key marker. The FT3/FT4 ratio reflects the peripheral thyroid hormone deiodination process and deiodinase activity, especially the decrease in the conversion of thyroxine (T4) to triiodothyronine (T3). Previous studies have found that the FT3/FT4 ratio is closely associated with the onset and adverse prognosis of CAD (Liu et al., 2022; Lang et al., 2023; Brozaitiene et al., 2016). It is worth noting that, compared to the individual measurement of FT3 or FT4, the FT3/FT4 ratio is more valuable in evaluating thyroid hormone metabolism.

Despite its promise in assessing general CAD, the FT3/FT4 ratio’s utility in acute settings remains uncertain. Specifically, its accuracy—compared with FT3 alone—in predicting coronary lesion severity in patients with acute coronary syndrome (ACS), as measured by the synergy between percutaneous coronary intervention (SYNTAX) score, is not well defined. Given the pressing need for reliable markers to guide ACS management, this study aimed to explore the relationship between the FT3/FT4 ratio and the SYNTAX score in ACS patients.

## Methods

### Enrolled population

This single-center, retrospective investigation specifically targeted patients who were diagnosed with ACS and underwent percutaneous coronary intervention (PCI) between July 2018 and December 2020 at the Third People’s Hospital of Chengdu (Sichuan Province, China).

**FIGURE 1:**
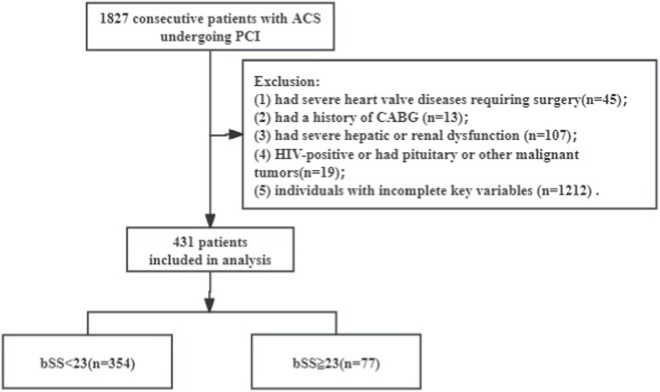
Flow chart for subject selection. ACS, acute coronary syndrome; PCI, percutaneous coronary intervention; CABG, coronary artery bypass grafting; bSS, baseline synergy between percutaneous coronary intervention score.

This study included 1827 participants aged between 26 and 97 years, among which 45 had severe heart valve diseases requiring surgery, 13 had a history of coronary artery bypass grafting (CABG), 107 had severe hepatic or renal dysfunction (creatinine clearance rate <15 mL/min), and 19 were HIV-positive or had pituitary or other malignant tumors; those with incomplete key variables (including baseline SYNTAX score (bSS) or THs, n = 1,212) were excluded. Finally, a cohort of 431 patients meeting the registration criteria was included in this analysis (Figure 1).

The research protocol and procedures were approved by the local Ethics Committee. The study adhered to the ethical guidelines outlined in the World Medical Association’s Declaration of Helsinki regarding human research, and strict confidentiality of personally identifiable patient data was maintained. Considering the retrospective nature of this study, written informed consent from patients was waived.

### Data collection and definitions

Information regarding the sociodemographic profiles, medical backgrounds, smoking and alcohol consumption patterns, symptoms and clinical indicators, laboratory findings, as well as medical and surgical details of the patients were extracted from electronic health records. The medical history included records of prior PCI, hypertension, diabetes, atrial fibrillation, stroke, and chronic obstructive pulmonary diseases. The conditions that are defined as ACS include unstable angina (UA), non-ST-segment elevation myocardial infarction (NSTEMI), and ST-segment elevation myocardial infarction (STEMI) (Bh et al., 2022).

### Collection of blood samples and determination of variables

Peripheral venous blood samples were obtained from all patients after overnight fasting (>8 h). THs, cardiac troponin T (CTnT), brain natriuretic peptide (BNP), fasting blood glucose (FBG), triglycerides (TG), total cholesterol (TC), low-density lipoprotein cholesterol (LDL-C), high-density lipoprotein cholesterol (HDL-C), serum creatinine clearance (Scr), and various other laboratory parameters were measured via standard biochemical techniques. The formula for computing the creatinine clearance (Ccr) in males is [(140 - age) × weight (kg)] ÷ [72 × Scr (mg/dL)], and for females, the outcome is multiplied by 0.85. The left ventricular ejection fraction (LVEF) was assessed via the two-dimensional modified Simpson method.

### Thyroxine, TSH, and calculation of the FT3/FT4 ratio

The standard reference intervals for FT3, FT4, and TSH were 2.63–5.70 pmol/L, 9.01–19.05 pmol/L, and 0.35–4.94 mU/L, respectively. The ratio of FT3 to FT4 served as a marker for peripheral thyroid hormone sensitivity.

### Assessment of coronary atherosclerotic severity

Each patient was diagnosed via coronary angiography (CAG) conducted by experienced cardiac specialists to pinpoint the location and extent of stenosis. CAD is defined as the narrowing of the diameter of at least one major epicardial coronary artery by more than 50%, whereas stenoses <50% in all coronary arteries are categorized as non-coronary heart disease. Moreover, the intricate nature of the entire coronary artery lesion was quantitatively assessed based on anatomical aspects such as lesion location, severity, bifurcation, and calcification. To ensure accuracy, the findings were verified by two independent researchers via an online tool (http://syntaxscore.com/) by calculating the SYNTAX score from preoperative angiography. The participants were classified into two groups: bSS ≥23 (77 cases) and bSS <23 (354 cases).

### Statistical analysis

Normality was assessed via the Kolmogorov-Smirnov test; continuous data were defined as means with standard deviations or medians with interquartile ranges, and were compared via Student’s t-test or the Mann-Whitney U test, respectively. Categorical data were presented as frequencies and percentages, and were compared via the chi-square test. For the evaluation of FT3/FT4 tertiles, continuous variables were subjected to one-way ANOVA, and the Kruskal-Wallis test was used for parametric and non-parametric data. The relationship between the FT3/FT4 levels and the severity of CAD determined by the angiography (bSS <23 vs. bSS ≥23) was examined via logistic regression analysis. Variables with a significance level of P < 0.05 in the univariate logistic regression analysis were incorporated into a multivariate model to ascertain the independent correlation between the FT3/FT4 ratio and bSS ≥23, quantified via the odds ratios (ORs) accompanied by a 95% confidence interval (CI). Model I was adjusted for age and body mass index (BMI), whereas Model II was adjusted for age, BMI, diastolic blood pressure (DBP), BNP, serum albumin (ALB), HDL-C, and Ccr. By calculating the area under the receiver operating characteristic (ROC) curve and performing a decision curve analysis (DCA), we aimed to assess the diagnostic accuracy of the FT3/FT4 levels in identifying the severity of CAD among patients with ACS. Restricted cubic splines (RCS) were utilized to examine the dose-response relationship between the FT3/FT4 ratio and the severity of CAD. Stratified analyses and testing were performed to assess the interaction between FT3/FT4 and bSS ≥23, considering variables such as the age (<65, ≥65 years), gender (male vs. female), smoking status (smoker vs. non-smoker), alcohol usage (drinker vs. non-drinker), glucose conditions (diabetes, prediabetes, normal glucose), hypertension (with vs. without), and multi-vessel disease (MVD, yes vs. no). For all analyses, a p value of <0.05 was considered statistically significant. The statistical analyses were conducted using IBM SPSS Statistics 29.0.1.0 (IBM Corporation, Armonk, NY, USA), R version 4.2.3, and GraphPad Prism 10.1.2 software.

## Results

### Baseline characteristics

The baseline characteristics of the enrolled patients are shown in Table 1. Among the 431 ACS patients (212 UA, 104 NSTEMI, and 115 STEMI), 74.0% were male, and the median age of the overall population was 69 (60, 77) years. Patients were stratified into three groups according to the tertiles of the FT3/FT4 ratio (T1, FT3/FT4 ≤0.27, n = 144; T2, 0.27 <FT3/FT4 ≤0.33, n = 144; T3, FT3/FT4 >0.33, n = 143). Compared with patients in the lowest tertile of the FT3/FT4 ratio (T1) group, patients in the T2 and T3 groups tended to be younger, have a lower incidence of hypertension and diabetes, and have a lower incidence of STEMI and cardiogenic shock. Moreover, the participants presented elevated heart rates along with higher levels of CTnT, BNP, FBG, and FT4. Conversely, the levels of albumin, FT3, the FT3/FT4 ratio, and LVEF were comparatively lower.

**TABLE 1:** The baseline characteristics based on tertiles of FT3/FT4 ratio.

Varies	Totel (n = 431)	T1 (n = 144)	T2 (n = 144)	T3 (n = 143)	P value
Male sex, n (%)	319 (74.0)	101 (70.1)	102 (70.8)	116 (80.6)	0.060
Age, years	69 (60, 77)	71 (61, 79)	69 (60.76)	65 (58, 73)	**0.005**
BMI, kg/m2	24.66 ± 3.29	24.67 ± 3.51	24.55 ± 3.05	24.77 ± 3.31	0.858
Smoking, n (%)	163 (37.82)	53 (36.8)	50 (34.7)	60 (42.0)	0.429
Alcohol, n (%)	63 (14.62)	14 (9.7)	23 (16.0)	36 (25.2)	0.109
Hypertension, n (%)	299 (69.4)	111 (77.1)	100 (69.4)	88 (61.5)	**0.017**
Diabetes mellitus, n (%)	114 (26.45)	46 (31.9)	46 (31.3)	23 (16.1)	**0.003**
Previous CHD, n (%)	82 (19)	21 (14.6)	34 (23.6)	27 (18.9)	0.149
Previous PCI, n (%)	30 (7)	6 (4.2)	12 (8.4)	12 (8.4)	0.272
Previous COPD, n (%)	16 (3.7)	6 (4.2)	6 (4.2)	4 (2.8)	0.778
Atrial fibrillation, n (%)	14 (3.2)	6 (4.2)	5 (3.5)	3 (2.1)	0.603
Stroke, n (%)	23 (5.3)	9 (6.3)	3 (2.1)	11 (7.7)	0.090
UA, n (%)	212 (49.2)	54 (37.5)	70 (48.6)	88 (61.5)	**<0.001**
NSTEMI, n (%)	104 (24.1)	47 (32.6)	34 (23.6)	27 (18.9)	0.093
STEMI, n (%)	115 (26.7)	47 (32.6)	40 (27.8)	28 (19.6)	**0.041**
SBP(mmHg)	133 (120, 148)	130 (116, 147)	135 (122, 154)	130 (120, 144)	0.052
DBP(mmHg)	77 (70, 88)	76 (69, 88)	79 (73, 89)	77 (69, 88)	0.265
Heart rate (bpm)	77 (67, 84)	78 (67, 87)	78 (68, 84)	74 (66, 83)	**0.019**
Cardiogenic shock (%)	12 (2.8)	9 (6.3)	2 (1.4)	1 (0.7)	**0.008**
Heart arrest (%)	1 (0.2)	1 (0.7)	0 (0)	0 (0)	0.368
Troponin-T (pg/mL)	27.1 (10.6, 290.4)	62.2 (17.8, 1592.0)	23.3 (10.5, 355.0)	18.0 (8.5, 83.6)	**<0.001**
BNP (pg/mL)	104.1 (41.3, 274.7)	209.9 (75.8, 697.2)	101.2 (41.5, 228.3)	63.5 (23.9, 124.3)	**<0.001**
Ccr (mL/min)	71.1 (54.3, 89.9)	63.2 (45.2, 86.8)	71.1 (53.9.90.1)	76.2 (59.2, 91.1)	**0.010**
FBG (mmol/L)	5.74 (5.01, 7.37)	6.42 (5.21, 8.37)	5.63 (5.01, 7.42)	5.33 (4.83, 6.38)	**<0.001**
Triglyceride (mmol/L)	1.47 (1.06, 2.03)	1.36 (1.03, 2.32)	1.45 (0.98, 1.91)	1.51 (1.18, 2.06)	0.226
Total cholesterol (mmol/L)	4.35 (3.51, 5.12)	4.19 (3.33, 4.98)	4.44 (3.55, 5.21)	4.38 (3.58, 5.19)	0.351
LDL-C (mmol/L)	2.62 (2.01, 3.27)	2.52 (1.91, 3.20)	2.68 (2.07, 3.27)	2.70 (2.05, 3.32)	0.515
HDL-C (mmol/L)	1.11 (0.95, 1.33)	1.08 (0.85, 1.28)	1.14 (0.99, 1.39)	1.10 (0.95, 1.29)	0.340
Albumin (g/L)	39.9 (36.9, 42.4)	37.9 (34.8, 41.5)	40.3 (37.2, 43.0)	40.5 (38.4, 43.6)	**<0.001**
TSH (mmol/L)	1.89 (1.13, 3.05)	1.70 (0.98, 2.75)	1.99 (1.23, 3.09)	1.95 (1.22, 3.35)	0.241
FT3 (pmol/L)	3.77 (3.31, 4.20)	3.20 (2.76, 3.54)	3.83 (3.51, 4.06)	4.34 (3.95, 4.76)	**<0.001**
FT4 (pmol/L)	12.51 (11.36, 13.68)	13.61 (12.48, 14.68)	12.56 (11.69, 13.59)	11.37 (10.54, 12.44)	**<0.001**
FT3/FT4 ratio	0.30 (0.26, 0.35)	0.24 (0.51, 0.56)	0.30 (0.28, 0.32)	0.37 (0.35, 0.41)	**<0.001**
LVEF (%)	57 (50, 61)	57 (48, 60)	58 (53, 62)	59 (55, 62)	**<0.001**

The groups were stratified by the tertiles of FT3/FT4 ratio (T1, FT3/FT4≤0.27; T2, 0.27<FT3/FT4≤0.33; T3, FT3/FT4>0.33). Data are presented as the mean ± SD, median (upper and lower quartiles) or number (%). Bold indicates P value <0.05; BMI, body mass index; CHD, coronary heart disease; PCI, percutaneous coronary intervention; COPD, chronic obstructive pulmonary disease; UA, unstable angina; STEMI, ST-segment elevation myocardial infarction; NSTEMI, non-ST-segment elevation myocardial infarction; SBP, systolic blood pressure; DBP, diastolic blood pressure; BNP, brain natriuretic peptide; Ccr, creatinine clearance; FBG, fasting blood-glucos; LDL-C, low-density lipoprotein cholesterol; HDL-C, high-density lipoprotein cholesterol; TSH, thyroid-stimulating hormone; FT3, free triiodothyronine; FT4, free thyroxine; LVEF, left ventricular ejection fraction. Significant P-values are bolded.

Furthermore, as shown in Table 2, patients with higher bSS values on CAG demonstrated lower rates of branch lesions and chronic total occlusion of coronary arteries, along with more frequent usage of diuretics and hypoglycemic agents upon discharge.

**TABLE 2:** The angiographic data based on tertiles of FT3/FT4 ratio.

Varies	Totel (n = 431)	T1 (n = 144)	T2 (n = 144)	T3 (n = 143)	P value
bSS	13 (8, 20)	16 (9, 23)	14 (7, 20)	10 (7, 18)	**<0.001**
MVD, n (%)	136 (31.6)	55 (38.2)	43 (29.9)	38 (26.6)	0.092
LM, n (%)	21 (4.9)	7 (4.9)	7 (4.9)	7 (4.9)	0.199
Branches lesions (%)	26 (6.0)	7 (4.9)	9 (6.3)	10 (7.0)	**0.009**
Calcified lesion s (%)	59 (13.7)	28 (19.4)	17 (11.8)	14 (9.8)	0.213
Thrombosis (%)	20 (4.6)	11 (7.6)	9 (6.3)	0 (0)	0.150
CTO (%)	73 (16.9)	23 (16.0)	23 (16.0)	27 (18.9)	**<0.001**
Long lesion (%)	136 (31.6)	59 (41.0)	46 (31.9)	31 (21.7)	0.829
Coronary rotablation (%)	7 (1.6)	4 (2.8)	1 (0.7)	2 (1.4)	0.365
Thrombus aspiration (%)	8 (1.9)	5 (3.5)	2 (1.4)	1 (0.7)	0.196
Number of stents	1 (1, 2)	1 (1, 2)	1 (1, 2)	1 (1, 2)	0.191
Length of stents (mm)	29 (20, 48)	33 (22, 56)	29 (20, 46)	29 (20, 46)	0.195

The groups were stratified by the tertiles of FT3/FT4 ratio (T1, FT3/FT4≤0.27; T2, 0.27<FT3/FT4 ≤0.33; T3, FT3/FT4>0.33). Data are presented as median (upper and lower quartiles) or number (%). Bold indicates P value <0.05; other abbreviations seen in Table 1. Significant P-values are bolded.

### Correlation between the FT3/FT4 ratio and SYNTAX score

**FIGURE 2:**
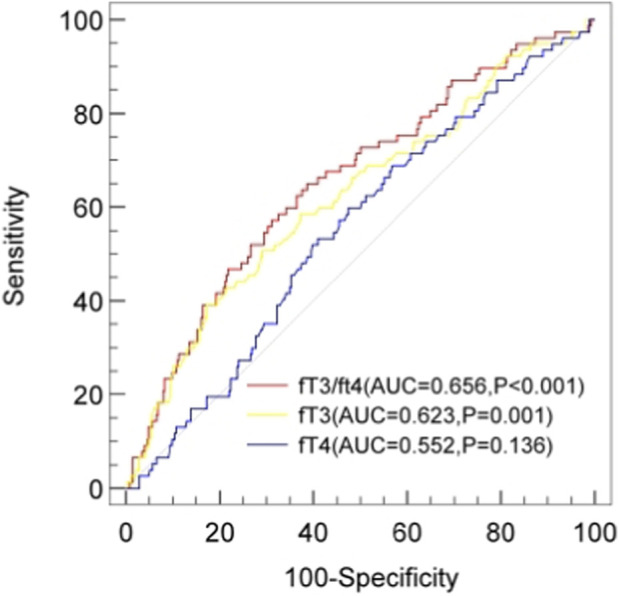
ROC curves for predicting a mid/high SYNTAX score. The area under the ROC curve of the FT3, FT4, and FT3/FT4 ratio for predicting a mid/high SYNTAX score (≥23) was 0.623 (95% CI 0.552–0.694; P = 0.001), 0.552 (95% CI 0.552–0.694; P = 0.001), and 0.656 (95% CI 0.587–0.724; P < 0.001), respectively. ROC, receiver operating characteristic; SYNTAX score, synergy between percutaneous coronary intervention score.

The ROC curves demonstrating the relationship between FT3, FT4, and the FT3/FT4 ratio with bSS were analyzed. The area under the ROC curve (AUC) showed that FT3/FT4 (AUC = 0.656, 95% CI: 0.587–0.724; P < 0.001) exhibited a superior discriminative ability compared with FT3 (AUC = 0.623, 95% CI: 0.552–0.694; P = 0.001) and FT4 (AUC = 0.552; 95% CI: 0.484–0.620; P = 0.136) (Figure 2).

**FIGURE 3:**
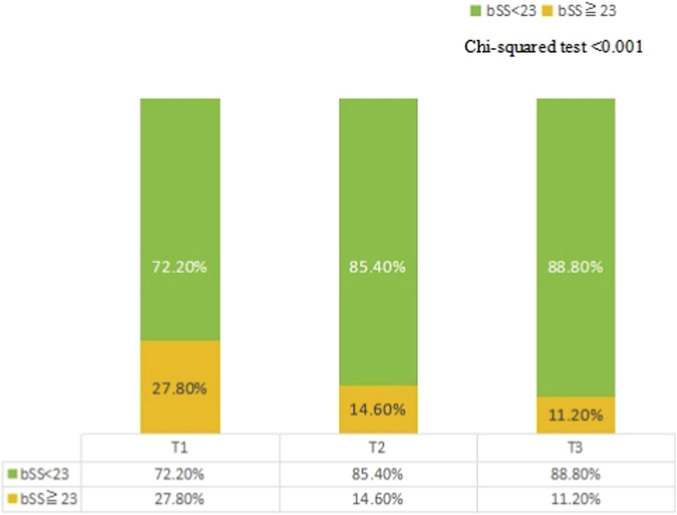
Comparison of the bSS according to the FT3/FT4 tertiles. The proportion of patients with bSS<23 and bSS≧23 in patients presenting with acute coronary syndrome stratified according to the tertiles of FT3/FT4. bSS, baseline synergy between percutaneous coronary intervention score.

**FIGURE 4:**
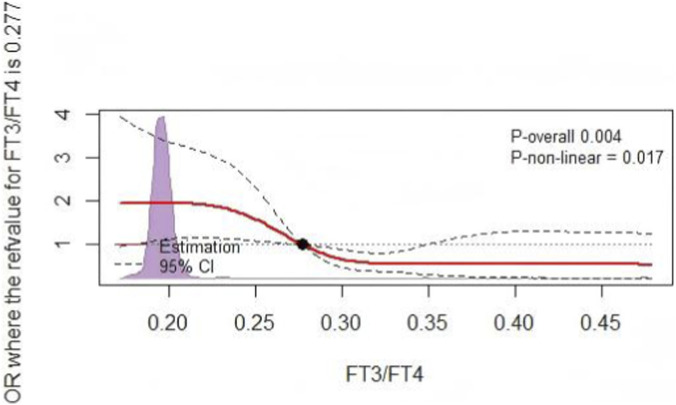
The RCS analysis. RCS for the odds ratio of a mid/high SYNTAX score. RCS, restricted cubic spline; SYNTAX score, synergy between percutaneous coronary intervention score.

Univariate logistic regression analysis identified age, BMI, DBP, Ccr, BNP, HDL-C, ALB, FT3, and FT3/FT4 as potential influencing factors (P < 0.05) for patients with a mid/high coronary artery lesions (bSS ≥23, Table 3). Based on the predictive factors identified via the univariate logistic regression, we developed a model for predicting mid/high SYNTAX scores, incorporating FT3/FT4 with FT3 as one of its components. To avoid potential interaction effects, FT3 was not included in the multivariable logistic regression model. The results indicated that, as a continuous variable, there was no significant difference in the occurrence of mid-/high-risk coronary anatomical complexity with respect to the FT3/FT4 ratio (P = 0.169). However, the FT3/FT4 ratio, as a three-category variable, remained an independent factor influencing the mid-/high-risk SYNTAX scores. Compared with the T1 group, the T3 group had a 0.47-fold lower risk of mid-/high-risk bSS (OR, 0.470; 95% CI: 0.227–0.971; P = 0.041) (Table 4). Compared to patients in the T1 group, the higher the stratification of FT3/FT4 ratio, the lower the proportion of mid-/high coronary anatomical complexity (SYNTAX score ≧23) risk (Figure 3). Moreover, through RCS analysis, a significant negative non-linear dose-response relationship was found between the FT3/FT4 ratio and SYNTAX score (non-linear P = 0.017), with an overall statistically significant association (P = 0.004) (Figure 4).

**TABLE 3:** Univariate and multivariate logistic regression analysis for predicting a mid/high SYNTAX score.

Variables	Univariate analysis			Multivariate analysis		
	OR	95% CI	P value	OR	95% CI	P value
Male sex, n (%)	1.185	0.665–2.112	0.565			
Age, years	1.036	1.012–1.061	**0.003**	0.993	0.958–1.030	0.717
BMI, kg/m^2^	0.920	0.852–0.994	**0.034**	0.928	0.840–1.025	0.141
Smoking, n (%)	0.927	0.556–1.546	0.771			
Alcohol, n (%)	0.848	0.410–1.751	0.655			
Hypertension, n (%)	1.431	0.814–2.516	0.213			
Diabetes mellitus (%)	1.139	0.658–1.917	0.642			
Previous CHD (%)	1.145	0.621–2.111	0.665			
Previous MI (%)	0.410	0.052–3.226	0.397			
Previous PCI (%)	0.914	0.338–2.468	0.859			
Previous COPD (%)	1.562	0.490–4.979	0.451			
Atrial fibrillation (%)	1.264	0.344–4.643	0.724			
Stroke (%)	2.112	0.838–5.326	0.113			
Chronic kidney diseases (%)	2.822	0.660–12.067	0.162			
SBP (mmHg)	0.994	0.982–1.005	0.994			
DBP (mmHg)	0.973	0.954–0.992	**0.007**	0.976	0.955–0.998	**0.033**
Heart rate (bpm)	1.005	0.988–1.023	0.560			
Troponin-T (pg/mL)	1.000	1.000–1.001	0.145			
Ccr (mL/min)	0.979	0.969–0.989	<0.001	0.984	0.968–1.000	0.056
BNP (pg/mL)	1.000	1.000–1.001	<0.001	1.000	1.000–1.001	0.240
FBG (mmol/L)	1.021	0.934–1.116	0.650			
Triglyceride (mmol/L)	0.949	0.763–1.181	0.640			
Total cholesterol (mmol/L)	0.988	0.922–1.059	0.733			
LDL-C (mmol/L)	1.053	0.803–1.380	0.709			
HDL-C (mmol/L)	0.400	0.165–0.971	**0.043**	0.248	0.087–0.710	**0.009**
Albumin (g/L)	0.932	0.879–0.987	**0.017**	1.016	0.943–1.095	0.668
TSH(mIU/L)	0.913	0.782–1.067	0.253			
FT3 (pmol/L)	0.459	0.322–0.656	**<0.001**			
FT4 (pmol/L)	0.992	0.940–1.046	0.759			
FT3/FT4 ratio tertiles						
T1	Ref.	Ref.	Ref.	Ref.	Ref.	Ref.
T2	0.444	0.246–0.800	**0.007**	0.496	0.244–1.008	0.053
T3	0.328	0.174–0.618	**<0.001**	0.470	0.227–0.971	**0.041**

Abbreviations as shown in Table 1. Significant P-values are bolded.

**TABLE 4:** Associations between FT3/FT4 ratio and complexity of ACS (Logistic regression models).

Models	Non-adjusted			Model I			Model II		
	OR	95% CI	P value	OR	95% CI	P value	OR	95% CI	P value
FT3/FT4 ratio	0.003	0.000–0.121	**0.002**	0.043	0.001–1.282	0.069	0.055	0.001–3.174	0.169
T1	Ref.			Ref.			Ref.		
T2	0.444	0.246–0.800	**0.007**	0.462	0.257–0.832	**0.010**	0.496	0.244–1.008	0.053
T3	0.328	0.174–0.618	**<0.001**	0.395	0.212–0.738	**0.004**	0.470	0.227–0.971	**0.041**

Non-adjusted, non-adjusted model.

Model I was adjusted for age and BMI.

Model II was adjusted for age, BMI, DBP, BNP, Albumin; HDL-C, Ccr.

Abbreviations as shown in Table 1. Significant P-values are bolded.

**FIGURE 5:**
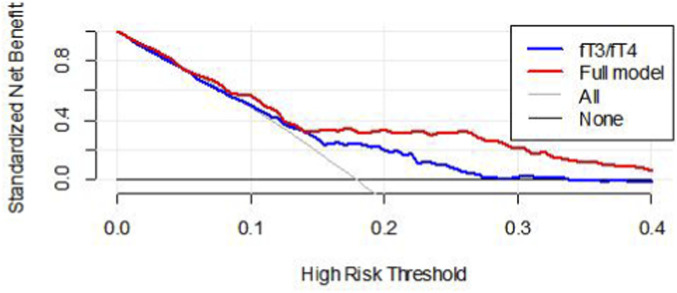
DCA of FT3/FT4 for the diagnostic nomogram. The net benefit calculated by adding true positive and minus the false positive corresponds to the measurement of Y-axis; X-axis represents the threshold probability. Full model was adjusted for age, BMI, DBP, BNP, Albumin, HDL-C, Ccr. DCA, decision curve analysis; BMI, body mass index; DBP, diastolic blood pressure; BNP, brain natriuretic peptide; HDL-C, high-density lipoprotein cholesterol; Ccr, creatinine clearance.

Finally, the utilization of DCA effectively addresses any limitations inherent in ROC curves by illustrating the proportions of false positives and true positives relative to the risk threshold. The graph demonstrates the clinical utility of the model based on the continuity of potential thresholds (x-axis) related to the risk of mid-/high-risk bSS and the net benefit (y-axis) of applying the model to risk-stratified patients, relative to the assumption that no patients would suffer from intermediate to high bSS. In this analysis, the FT3/FT4 ratio showed good predictive performance for mid-/high-risk bSS in clinical practice. Compared to using the FT3/FT4 ratio alone, the model combining the FT3/FT4 ratio with age, BMI, DBP, HDL-C, and albumin demonstrated greater net benefits within the range of intermediate to high-risk bSS (Figure 5).

### Subgroup analysis

**FIGURE 6:**
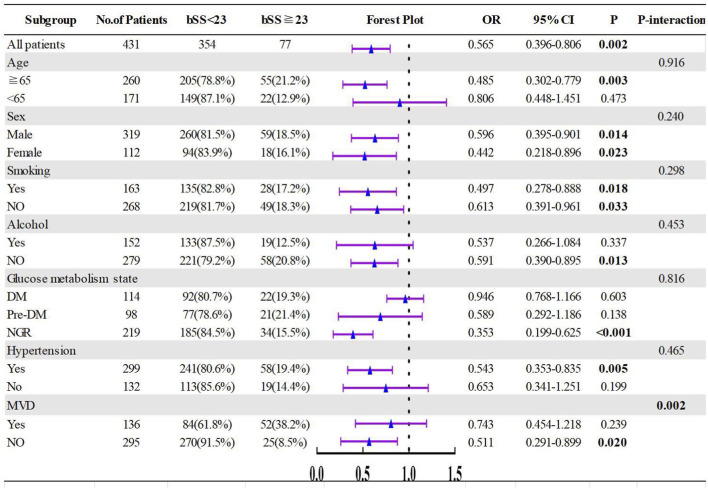
Subgroups analyses of FT3/FT4 ratio for predicting the SYNTAX score. DM, diabetes mellitus; Pre-DM, pre-diabetes mellitus; NGR, normal glucose regulation; MVD, multivessel disease.

Various subgroup analyses were conducted to assess the consistency of the predictive value of FT3/FT4 across patients with different demographic characteristics and comorbidities. The results indicated that FT3/FT4 ratio shows significant differences in predicting intermediate to high-risk coronary anatomical complexity in patients aged ≥65 years (OR = 0.485, 95%CI: 0.302–0.779, P = 0.003), non-drinkers (OR = 0.591, 95%CI: 0.390–0.895, P = 0.013), those with normal blood sugar levels (OR = 0.353, 95%CI: 0.199–0.625, P < 0.001), hypertension (OR = 0.543, 95%CI: 0.353–0.835, P = 0.005), and non-MVD patients (OR = 0.511, 95%CI: 0.291–0.899, P = 0.020). However, no significant differences were observed in predicting intermediate to high-risk coronary artery disease in patients aged <65 years (OR = 0.806, 95%CI: 0.448–1.451, P = 0.473), drinkers (OR = 0.537, 95%CI: 0.266–1.084, P = 0.337), those with diabetes (OR = 0.946, 95%CI: 0.768–1.166, P = 0.603), prediabetes (OR = 0.589, 95%CI: 0.292–1.186, P = 0.138), non-hypertension (OR = 0.653, 95%CI: 0.341–1.251, P = 0.199), and MVD patients (OR = 0.743, 95%CI: 0.454–1.218, P = 0.239). Significant differences were observed in male (OR = 0.485, 95%CI: 0.302–0.779) and female (OR = 0.485, 95%CI: 0.302–0.779) patients, as well as smokers (OR = 0.485, 95%CI: 0.302–0.779) and non-smokers (OR = 0.485, 95%CI: 0.302–0.779). The above indicators showed statistical significance in the interaction test within MVD (interaction P < 0.05), while there were no statistical differences in the interaction tests for the remaining indicators (all interaction P values >0.05) (Figure 6).

## Discussion

In this retrospective study, we found that (1) The reduced FT3/FT4 ratio shows a significant negative correlation with the severity of coronary lesions in patients with ACS. (2) A non-linear dose-response relationship exists between FT3/FT4 levels and the SYNTAX score. (3) The predictive value of FT3/FT4 for the severity of CAD is significantly valuable in the elderly (age ≧65 years), individuals with good metabolic status (normal blood sugar, non-drinkers), and those with hypertension. Regardless of gender or smoking status, the FT3/FT4 ratio is an important predictive indicator for mid-/high coronary anatomical complexity.

This study not only provides a new perspective regarding the management strategies for coronary heart disease, but also serves as a catalyst for a deeper exploration of the underlying molecular biological mechanisms. Although the detailed mechanisms explaining the link between FT3/FT4 and cardiovascular diseases remain are not completely elucidated, THs have been reported to exert direct effects on myocardial cells, regulate lipid metabolism, maintain glucose homeostasis, and influence blood pressure, thereby impacting the development of cardiovascular diseases (Jabbar et al., 2016; Taddei et al., 2003; Cai et al., 2011; Sinha et al., 2018; Rasool et al., 2023). THs have direct anti-atherosclerotic effects through vasodilation, production of vasodilatory molecules, inhibition of angiotensin II receptor expression, and signaling, with thyroid hormone-induced vasodilation involving nitric oxide synthase through the PI3K/Akt pathway, AT1R signal inhibition, and increased vascular smooth muscle K+ channel activity (Gerdes, 2015). Study indicated that bolus injections of T3 or T4 in rats trigger transient, dose-dependent coronary perfusion pressure reduction and increased arterial vasodilation, suggesting a direct non-genomic effect of THs in inducing coronary artery dilation to prevent myocardial ischemia (Yoneda et al., 1998). Hypothyroidism impairs coronary blood flow and is associated with vascular remodeling, but treatment with THs in subclinical hypothyroid hamsters can restore impaired coronary blood flow to normal (Khalife et al., 2005). Hypothyroidism is linked to atherosclerosis due to dyslipidemia (elevated LDL cholesterol, decreased HDL cholesterol, and increased lipoprotein-a (Staels et al., 1990; Kung et al., 1995)), diastolic hypertension (increased arterial stiffness (Obuobie et al., 2002)), endothelial dysfunction, as well as elevated homocysteine levels (Guthikonda and Haynes, 2006), high C-reactive protein levels, and elevated plasminogen activator inhibitor-1 levels, exacerbating or leading to atherosclerosis and ischemic heart disease (Ichiki, 2010). Studies by Sato et al. show that physiological concentrations of T3 (15 pmol/L FT3) increased matrix Gla protein mRNA levels threefold in rat aortic smooth muscle cells and cultured human coronary artery smooth muscle cells. Since matrix Gla protein is considered a potent inhibitor of vascular calcification, it is speculated that THs may prevent vascular calcification associated with atherosclerotic plaque progression (Ichiki, 2010). Inadequate angiogenesis is one of the causes of tissue ischemia, with T3 inducing myocardial angiogenesis through PDGF/Akt, αVβ3 integrin MAPK, FGF2, and TRβ mediation (Gerdes, 2015; Makino et al., 2009). On the other hand, angiogenesis within atherosclerotic plaques is thought to render the plaque unstable, possibly leading to plaque rupture and subsequent thrombus formation, a major mechanism of ACS (Jain et al., 2007). And the renin-angiotensin system plays a crucial role in accelerating atherosclerosis, with reports indicating that T3 increases renin production and secretion (Resnick and Laragh, 1982). Excess thyroid hormone levels also affect atherosclerosis. Hence, maintaining normal thyroid function is beneficial for preventing the progression of atherosclerosis.

The FT3/FT4 ratio is recognized as a valuable marker associated with both thyroid hormone resistance and cardiovascular diseases. Consequently, the prompt identification and management of abnormal fluctuations in FT3/FT4 levels among patients with coronary heart disease is critical. The main pathophysiological mechanism underlying FT3/FT4 alterations may involve reduced 5′-deiodinase activity, which impacts the conversion of T4 to T3. The thyroid hormone receptor has a 10-fold greater affinity for T3 than for T4 (Jabbar et al., 2016; Liu et al., 2022). Given the presence of thyroid hormone receptors in myocardial and vascular endothelial tissues, even slight variations in FT3/FT4 levels may be associated with the severity of CAD in individuals with normal thyroid function. While an association between lower FT3/FT4 ratios and intermediate to high-risk SYNTAX has been observed, it is important to note that various acute conditions and medications can also influence thyroid hormone levels. Even though this study made multiple model adjustments, the potential confounding effects of these factors cannot be excluded. Therefore, larger-scale, multicenter, prospective studies are needed to explore the relationship between the FT3/FT4 ratio and the severity of coronary artery atherosclerosis.

The Gensini and SYNTAX scores are commonly used to assess the complexity and severity of CAD (Boyraz and Peker, 2022). The Gensini score primarily assesses plaque burden but does not involve features such as bifurcation, calcification, and tortuosity; while, the SYNTAX score reflects the type of plaque and the complexity of PCI, providing guidance relative to the anatomical structure of CAD, as well as the best treatment strategies for high-risk patients by clinical physicians. Prior research indicates that the SYNTAX score outperforms the Gensini score in prognosticating outcomes for patients with CAD. Moreover, decisions based on the SYNTAX score align more closely with those of the cardiac team compared to decisions relying on the Gensini score (Boyraz and Peker, 2022; Kalkan et al., 2018). Emerging epidemiological evidence indicates a direct link between decreased FT3/FT4 levels and an elevated risk of cardiovascular disease in the general population. Daswani et al. previously utilized the Gensini scoring system to assess the severity of CAD and reported that FT3 levels were negatively correlated with the severity of coronary arteriosclerosis (Daswani et al., 2015). Auer et al. found that higher levels of serum-free thyroid hormone concentrations were associated with decreased severity of coronary atherosclerosis, and higher levels of serum thyrotropin concentrations were associated with increased severity of coronary atherosclerosis (Auer et al., 2003). Regardless, they did not investigate the relationship between the FT3/FT4 levels and the severity of coronary anatomical complexity. To the best of our knowledge, this study is the first to employ the SYNTAX score to confirm an independent inverse relationship between FT3/FT4 and coronary-artery severity among ACS patients. The results revealed that individuals with lower FT3/FT4 ratios are typically older, present higher incidences of hypertension and diabetes, lower albumin levels, reduced cardiac function, and demonstrate more widespread and intricate coronary artery anatomical abnormalities. These results suggest that FT3/FT4, as an unconventional CAD risk factor, can influence the progression of coronary atherosclerosis, either directly or indirectly. However, since CAD itself can also lead to changes of thyroid hormone levels (Telkova and Tepliakov, 2004), the current research cannot establish a causal relationship. Future higher-quality clinical and basic research is needed to reveal the causal relationship between thyroid hormone sensitivity and the severity of CAD. Through genomics, metabolomics, and other in-depth studies, the molecular mechanisms of the interaction between the FT3/FT4 ratio and CAD need to be explored.

This study found that patients with higher FT3/FT4 ratio tertiles had lower SYNTAX scores. Although no significant associations were observed in terms of MVD, calcified lesions, thrombotic events, diffuse long lesions, and thrombus aspiration, the trend still suggested that higher FT3/FT4 tertiles corresponded to lower risks of certain vascular lesions. However, as FT3/FT4 tertiles increased, the proportion of bifurcation lesions and CTO lesions also increased, showing a significant positive correlation. These results reveal that the relationship between thyroid hormones and the complexity of coronary artery disease is not a simple linear one but rather a complex relationship with lesion-specific characteristics. A higher FT3/FT4 ratio (reflecting higher T4 to T3 conversion efficiency) has a mixed impact on coronary artery disease. Higher FT3/FT4 ratio signifies more active thyroid function and metabolism, which may be associated with more favorable lipid profiles (such as lower cholesterol), better insulin sensitivity, and healthier endothelial function. These factors together may slow down the overall progression of atherosclerosis, resulting in a lighter overall burden of coronary artery disease (lower SYNTAX score). Additionally, adequate FT3 has a direct positive inotropic effect on myocardial cells, maintaining better cardiac function, and partially inhibiting the progression of diffuse lesions through effective blood flow flushing and improved microcirculation. Bifurcation and CTO lesions are the most challenging lesion types in interventional therapy, with their formation mechanisms closely related to local hemodynamics and mechanical stress, in addition to systemic atherosclerosis factors (Zuin et al., 2024)^[37]^. Higher FT3 levels significantly increase shear stress on the vessel wall, and blood flow turbulence and low shear stress at bifurcations and certain segments prone to CTO (such as curved vessels) are already hotspots for damage due to increased blood impact force, which continuously damages the endothelium in these specific areas, accelerating plaque formation and progression. Moreover, higher T3 levels may promote differentiation of vascular smooth muscle cells, making it easier to induce calcification and pathological vascular remodeling at bifurcations, ultimately leading to complex CTO lesions. This discovery highlights the importance of not only assessing thyroid function as “normal” or looking solely at overall scores (such as the SYNTAX score) when evaluating coronary heart disease risk. For patients with higher FT3/FT4 ratios, even if the overall burden is not severe, it is crucial to remain vigilant for the presence of hidden “killer lesions” (such as bifurcation lesions and CTO), as these lesions could pose significant cardiovascular event risks.

Additionally, the study also found that in the subanalysis, the decrease in the ratio of FT3/FT4 was significantly associated with a higher risk of CAD in individuals aged ≥65, non-drinkers, those with normal blood glucose levels, hypertension patients, and individuals without MVD (OR<1, suggesting a protective effect). However, in individuals aged <65, drinkers, those with diabetes or impaired glucose tolerance, individuals without hypertension, and those with MVD, the FT3/FT4 ratio showed no significant predictive value. This indicates that the predictive value of the FT3/FT4 ratio for CAD is not universal, but rather dependent on the specific metabolism and clinical background of the patients. The elderly population (≥65 years), individuals with good metabolic status (normal blood glucose, non-drinkers), hypertension patients, and those without MVD represent a “relatively pure” aging or hypertension-related atherosclerosis model. Their risk of CAD primarily comes from factors such as aging and hypertension, without being severely affected by stronger and more complex metabolic disorders such as diabetes, alcohol, or existing MVD. In these patients, maintaining a higher FT3/FT4 ratio may signify better metabolic health (improved lipid metabolism, healthier weight, and less insulin resistance tendency), as well as better vascular function (more effective NO-mediated vasodilation and endothelial protection). Therefore, in these relatively “pure” pathophysiological backgrounds, the positive metabolic and cardiovascular effects of thyroid hormones are effectively realized, showing a significant protective effect. On the other hand, individuals aged <65, drinkers, those with diabetes or impaired glucose tolerance, individuals without hypertension, and those with MVD represent a “high metabolic disorder burden” or “late-stage disease” status. The mechanisms driving CAD in younger patients may involve more complex genetic, inflammatory, or immune factors, different from those in elderly patients, thus resulting in different responses to thyroid hormone status. Due to the strong atherosclerotic factors of diabetes and alcohol consumption themselves, their damaging effects on blood vessels and disruption of metabolism may “overwhelm” or “mask” the relatively subtle protective effects of thyroid hormones. Since hypertension itself is a core risk factor for coronary heart disease, in a group where hypertension is the main contradiction, having a higher FT3/FT4 ratio (indicating relatively healthy metabolic conversion ability) may offset some of the risks associated with hypertension, thus showing a protective effect. Conversely, in the subgroup without hypertension, coronary heart disease may be caused by other more dominant mechanisms (such as genetic factors, hypercoagulability, undetected metabolic issues), making the role of the FT3/FT4 ratio not significant. For patients with MVD, the atherosclerotic process is already widespread and severe, and subtle differences in thyroid hormone status may have limited effects on reversing or significantly influencing this advanced disease progression, leading to a weakened predictive role of the FT3/FT4 ratio. These results suggest that the interaction between thyroid hormones and the cardiovascular system is deeply regulated by glucose metabolism, alcohol intake, and the overall degree of microvascular disease, and future research should focus on elucidating the molecular mechanisms underlying these interactions.

Currently, in the latest developments in the diagnosis and treatment of CAD, the ratio of FT3/FT4 has not yet been integrated into existing risk prediction tools (such as the GRACE score or TIMI risk score). Given that this parameter can be obtained through early blood tests, in clinical practice, perhaps the FT3/FT4 ratio could be integrated into the risk assessment models for specific populations (such as the elderly non-diabetic population). This initiative may improve the early predictive accuracy for middle to high-risk CAD patients.

## Limitations

This study has several limitations. First, it is a single-center study, and the baseline data and CAG data primarily come from electronic health records. Due to restrictions in China’s medical insurance policies, many patients did not undergo FT3 and FT4 testing, leading to the exclusion of a large number of patients due to missing key baseline data. Although we compared the included and excluded cases and found no significant differences in demographic characteristics and comorbidities (refer to Supplementary Table S1 for details), residual confounding factors may still have an impact despite controlling for potential confounders in the models. Second, thyroid function assessment was only conducted once before CAG, lacking continuous monitoring, which means transient fluctuations in thyroid hormone levels due to acute illnesses and medication were not considered, potentially amplifying the correlation between FT3/FT4 ratio and syntax score. Third, our assessment focused solely on FT3 and FT4 levels, which represent direct indicators of thyroid function, without including measurements of total T3, total T4, or reverse T3 levels, or calculating SPINA-GD, which is potentially relevant to the SYNTAX scores. Fourth, this retrospective study exclusively enrolled ACS patients who underwent successful PCI rather than being randomized. These factors may introduce a certain selection bias and limit the generalizability of the study results to a broader population. Therefore, future research needs to be validated through prospective, multicenter, large-scale randomized studies.

Furthermore, this study selected the upper third percentile (the highest 33%) of the FT3/FT4 ratio as the threshold. This approach, based on statistical considerations rather than relying on pre-existing clinical diagnostic thresholds, was determined by the distribution characteristics of the study population to identify subtle variations in thyroid function within the traditionally considered normal range that still hold prognostic value. However, due to their high population specificity, methodological dependence, and relative nature, these specific thresholds cannot be directly generalized to other populations. In clinical practice, we must still rely on standardized laboratory reference ranges and make comprehensive judgments by integrating patients’ overall clinical manifestations.

## Conclusion

The FT3/FT4 ratio serves as an independent predictive factor for mid-/high-risk lesions in the coronary arteries of patients with ACS. Given that CAD requires comprehensive clinical management, performing standard thyroid hormone testing prior to coronary angiography and calculating the FT3/FT4 ratio to evaluate early perturbations in thyroid hormone homeostasis may facilitate the early identification of mid-/high-risk patients and the development of individualized prevention and treatment strategies.

## Data Availability

Publicly available datasets were analyzed in this study. This data can be found here: The datasets used and/or analyzed in the study are available from the corresponding authors upon reasonable request.

## Glossary

THs: thyroid hormones
CAD: coronary artery disease
FT3: free triiodothyronine
FT4: free thyroxine
T4: thyroxine
T3: triiodothyronine
ACS: acute coronary syndrome
SYNTAX score: synergy between percutaneous coronary intervention score
CABG: coronary artery bypass grafting
PCI: percutaneous coronary intervention
UA: unstable angina
STEMI: ST-segment elevation myocardial infarction
NSTEMI: non-ST-segment elevation myocardial infarction
CTnT: cardiac troponin T
BNP: brain natriuretic peptide
FBG: fasting blood glucose
TG: triglycerides
TC: total cholesterol
LDL-C: low-density lipoprotein cholesterol
HDL-C: high-density lipoprotein cholesterol
Scr: serum creatinine clearance
Ccr: creatinine clearance
LVEF: the left ventricular ejection fraction
CAG: coronary angiography
ORs: odds ratios
CI: confidence interval
BMI: body mass index
DBP: diastolic blood pressure
ALB: albumin
ROC: the receiver operating characteristic
DCA: decision curve analysis
RCS: restricted cubic splines
MVD: multi-vessel disease
AUC: the area under the ROC curve
