# Supplementary material for: The predictive value of the FT3/FT4 ratio for the severity of coronary artery disease in patients with acute coronary syndrome
Source: Front Physiol. 2025 Dec 10;16:1696692. doi: 10.3389/fphys.2025.1696692 (PMC12727579; doi:10.3389/fphys.2025.1696692)
Supplement: Supplementary file 1 [file Table1.docx]

**Table S1 Comparison of baseline characteristics between excluded cases and included cases.**

| **Varies** | **Total**  **（n=1827）** | **Include cases (n=431)** | **Exclude cases (n=1396)** | **P value** |
| --- | --- | --- | --- | --- |
| Male, n (%) | 1301（71.2） | 319 (74.0) | 982（70.3） | 0.160 |
| Age, years | 68（60.76） | 69 (60, 77) | 68（61, 75） | 0.177 |
| Current smoking, n (%) | 638（34.9） | 163 (37.8) | 475（34.0） | 0.243 |
| Alcohol, n (%) | 513（28.1） | 152(35.3) | 361（25.9） | 0.926 |
| Hypertension, n (%) | 1244（68.1） | 299（69.4） | 945（67.6） | 0.838 |
| Diabetes mellitus, n (%) | 546（29.9） | 114（26.5） | 432（30.9） | 0.071 |
| Previous CHD, n (%) | 343（18.8） | 82（19.0） | 261（18.7） | 0.856 |
| Previous PCI, n (%) | 161（8.8） | 30（7.0） | 131（9.4） | 0.121 |
| Previous COPD, n (%) | 57（3.2） | 16（3.7） | 41（2.9） | 0.346 |
| Atrial fibrillation, n (%) | 64（3.5） | 14（3.2） | 50（3.6） | 0.881 |
| Stroke, n (%) | 86（4.7） | 23（5.3） | 63（4.5） | 0.516 |
| Chronic kidney diseases, n(%) | 50（2.7） | 8（1.9） | 42（3.0） | 0.238 |
